# The impact of virtual reality exercise programs on postpartum pelvic pain and disability among women with lumbopelvic pain

**DOI:** 10.7717/peerj.21202

**Published:** 2026-07-01

**Authors:** Javeria Aslam, Alba Paris-Alemany, Raquel Díaz-Meco-Conde, Carlos Romero-Morales

**Affiliations:** 1 Faculty of Medicine, Health and Sports, Universidad Europea de Madrid, Villaviciosa de Odón, Madrid, Spain; 2 Department of Doctor of Physical Therapy/Shalamar School of Allied Health Sciences, Shalamar institute of Health Sciences, Lahore, Pakistan; 3 Department of Basic Health Sciences, Health Science Faculty, Universidad Rey Juan Carlos, Alcorcon, Madrid, Spain; 4 Motion in Brains Research Group, CSEU La Salle, Universidad Autónoma de Madrid, Madrid, Spain; 5 Instituto de Dolor Craneofacial y Neuromusculoesquelético (INDCRAN), Madrid, Spain

**Keywords:** Virtual reality, Postpartum rehabilitation, Lumbopelvic pain, Pelvic floor dysfunction, Disability, Fully immersive exercise environment, Traditional exercises, Pain management, Pelvic pain

## Abstract

**Background:**

Postpartum lumbopelvic pain (LPP) and pelvic floor dysfunction are highly prevalent conditions that contribute to persistent disability, pain, and impaired quality of life due to pelvic girdle pain. While conventional rehabilitation including traditional exercise (TE) methods such as pelvic floor muscle training and resistance exercises remains standard, they are often limited by poor adherence and reduced patient motivation. Virtual reality (VR) provides an innovative, non-invasive rehabilitation strategy, offering immersive and gamified environment that may improve patient engagement, neuromuscular control, and treatment outcomes.

**Objective:**

To compare the effectiveness of VR-based rehabilitation with TE therapy in reducing pain, disability, and pelvic floor dysfunction among postpartum women with LPP.

**Methods:**

A single-blind randomized controlled trial was conducted, including 60 postpartum women aged 20–40 years experiencing LPP were enrolled between 2 and 12 weeks after delivery. No participants beyond this window were included. The assignment followed a simple randomization process and was done computational with equal distribution (1:1) of the VR and TE group. An independent researcher created 60 sequentially numbered, opaque and sealed envelopes. After participant enrollment, each envelope was opened to determine group assignment, ensuring equal group sizes. Both groups completed 8 sessions of pelvic floor muscle training over one month. The VR group additionally performed immersive tasks using Meta Quest 2 headsets and Lumbar Pain Rehab software. Outcomes were assessed at baseline, 4th week and 8th week assesment using the Oswestry Disability Index (ODI), Visual Analog Scale (VAS), Pelvic Girdle Questionnaire (PGQ), McGill Pain Questionnaire (SF-MPQ), Pelvic Floor Distress Inventory (PFDI-20) and Simulator Sickness (SSQ). Following approval from the Institutional Review Board of Shalamar Medical and Dental College (SMDC), which is a constituent body of Shalamar, with IRB no.0543 and reference no. SMDC-IRB/AI/18-1/2023.

**Results:**

Groups were comparable at baseline. At the 4th week assesment, the VR group showed significantly greater improvements in pain intensity (Visual Analog Scale (VAS), *p* = 0.002), affective pain scores (SF-MPQ, *p* = 0.009), and pelvic girdle pain (PGQ, *p* ≤ 0.001). By the 8th week assesment, the VR group maintained superior outcomes in ODI (*p* = 0.003), VAS, SF-MPQ & PGQ (*p* < 0.001). Both groups achieved substantial improvement in patient-reported prolapse symptoms, with similar recovery trends in urinary and bowel dysfunction.

**Conclusion:**

VR-based rehabilitation demonstrated greater short–term effectiveness than TE in reducing disability, alleviating pain related to pelvic girdle. Its immersive design promotes adherence and engagement, making it a clinically valuable intervention for postpartum recovery, particularly in resource-limited settings.

**Trial registration:**

ClinicalTrials.gov Identifier: NCT05921747

## Introduction

Lumbopelvic pain (LPP) refers to self-reported discomfort in the lower back, sacroiliac joints, or a combination of these areas in pregnant and postnatal women (Goossens et al., 2021). This pain can extend to the posterior thigh, and it may or may not be accompanied by symphysis pubis pain. A considerable percentage of pregnant women stated that they had experienced LPP during or after delivery. While most women recover within three months postpartum, a significant proportion continue to experience pain, with prevalence rates ranging from 26.5% to 91.0% two to three years after delivery (Demilew et al., 2024).

Postpartum LPP arises from a combination of physiological and biomechanical factors. The ligamentous laxity and the redistribution of loads *via* the pelvic girdle and lumbar spine are the results of hormonal changes in pregnancy, including the elevation of relaxin (Aldabe et al., 2012). The presence of weakened core and pelvic floor muscles, residual instability with the pelvis, and postpartum asymmetry are also contributory factors that lead to persistent pain. Some of the risk factors for this pain include the maternal age, parity, body mass index, education level, and the hard working conditions (Aldabe et al., 2012). Postpartum female experiencing pelvic girdle pain (PGP) and low back pain (LBP) often finds it difficult to perform daily life activities, as confirmed by Vermani, Mittal & Weeks (2010).

Functional improvement in patients with LPP was found to be achieved through stabilization exercises that involve dynamic control of the lumbar segments and the pelvic joints (Ehsani et al., 2020). It is well-established that the coordination of muscle activity around the lumbopelvic region is vital to the generation of mechanical spinal stability (Zambarano, 2019). Prophylactic and therapeutic pelvic health includes the regular use of pelvic floor muscle training (PFMT), including kegel exercises. Evidence indicates that PFMT promotes increased muscular tone and endurance, which is instrumental in preventing and alleviating pelvic organ prolapse and urinary and fecal incontinence by enhancing urethral and anal sphincter closure mechanisms (Sheng et al., 2022). Moreover, a developed pelvic floor is a source of lumbopelvic stability, which prevents the risk of injury during physical efforts as well as alleviating pain related to dysfunction, especially after delivery. The cumulative effect is a significant improvement in core function, sexual health, and overall quality of life (Nimbi et al., 2020).

New advancements in technologies such as virtual reality (VR) have shown promising results for treating a variety of diseases and disorders, such as chronic pain. The major two mechanisms involved in VR analgesia are distraction and immersion. Distraction therapy can be used to reduce pain by directing the attentional resources out of the nociceptive signals when playing VR games. Immersive VR and virtual embodiment in particular is helpful in managing chronic pain, creating a sense of ownership over a virtual body in the first person perspective (Matamala-Gomez et al., 2019). This embodiment activates corresponding somatosensory and premotor cortices, which have been demonstrated to improve the pain-free range of motion in conditions like chronic unilateral shoulder pain (Álvarez de la Campa Crespo et al., 2023). In chronic neck pain, Gao showed that patients receiving VR therapy combined with conventional rehabilitation experienced significantly greater reductions in pain and disability compared to conventional therapy alone, with improvement maintained at 3-month follow-up (Guo et al., 2024). Systematic reviews and meta-analyses have demonstrated that VR interventions can significantly reduce pain in musculoskeletal disorders, particularly knee pain, though heterogeneity remains a limitation across studies (Zitti et al., 2025). Substantial evidence has already shown VR to be a plausible intervention in the management of burn pain, acute pain, and experimentally induced pain (Ahmadpour et al., 2019). Despite these promising findings in general musculoskeletal conditions and chronic pain populations, the application of VR specifically for postpartum LPP has not been adequately studied. While VR has been evaluated for acute procedural pain and in diverse chronic musculoskeletal settings, evidence focusing on postpartum females with LPP is lacking (Özyurt et al., 2025).

This is a major gap in the literature, considering the high prevalence of postpartum LPP and the weaknesses of the available treatments addressing the persistent pain and disability. Thus, the current research problem focuses on assessing the performance of a VR based intervention on primary pain outcomes and secondary disability outcomes in postpartum women with LPP to address a significant clinical gap.

## Material and Methods

### Study design

This was a single-blind randomized controlled trial that involved 60 postpartum women between the ages of 20 and 40 years old with LPP. The subjects were recruited based on the inclusion criteria.

**Randomization:** The assignment followed a simple randomization process and was done computationally with equal distribution (1:1) of the VR and traditional exercise (TE) group. In order to guarantee allocation concealment, an independent researcher created 60 sequentially numbered, opaque, and sealed envelopes that had group assignments. After participant enrollment, each envelope was opened to determine group assignment, ensuring equal group sizes.

**Figure 1:**
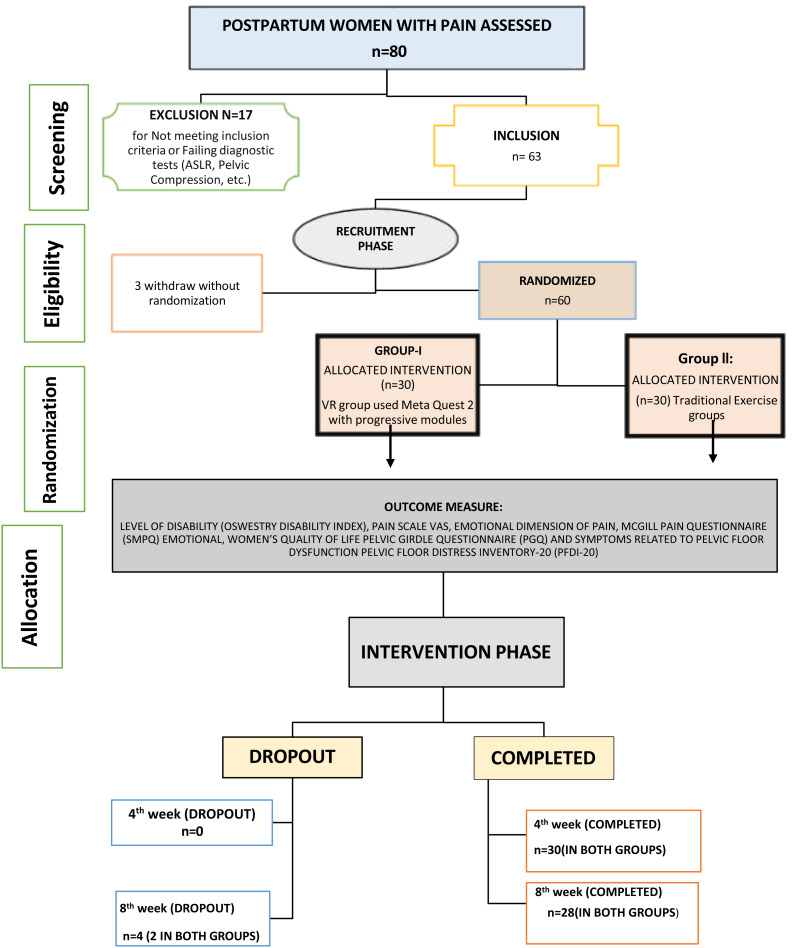
Flowchart of postpartum women with lumbopelvic pain through screening, randomization, intervention, and follow-up.

**Blinding:** Both outcome assessors and data analysts were blinded to group assignment during the research. Participants and treating therapists could not be blinded due to the nature of the interventions shown in Fig. 1.

### Ethical considerations

Ethical approval from the Institutional Review Board of Shalamar Medical and Dental College (SMDC), which is a constituent body of Shalamar Institute of Health Sciences, with IRB no. 0543 and reference no. SMDC-IRB/AI/18-1/2023. Written informed consent was obtained from all participants prior to enrollment. The study was conducted at the Gynecology department of Shalamar Hospital, Lahore, over 12 months from July 2023 to July 2024. This research was registered in ClinicalTrials.gov (Registration No. NCT05921747).

### Sample size calculation

The primary outcome of the trial was pain intensity, measured using the Visual Analog Scale (VAS). As no previous studies have assessed VR interventions for postpartum LPP, the sample size was estimated based on pain scores reported by Saleh, Botla & Elbehary (2019), who evaluated conventional core stability exercises in a similar population (mean ± SD: 32.35 ± 12.51). Using the WHO sample size calculator with 95% confidence and 95% power, a minimum of 17 participants per group was required. The sample size was expanded to 30 participants in each group to cover the possibility of dropouts with a total of 60 participants. Secondary outcomes were assessed to explore additional effects of the interventions. The study was not powered to detect significant differences in these secondary outcomes, and results should be interpreted as exploratory.

### Participants

A total of 60 postpartum women aged 20–40 years experiencing LPP were enrolled between 02 and 12 weeks after delivery. No participants beyond this window were included. The participants were identified after meeting established inclusion criteria and were drawn in simple random sampling.

#### Inclusion criteria

Inclusion criteria required a positive result in at least three of six clinical tests—Active Straight Leg Raise (ASLR), Pelvic Compression Test, Pelvic Distraction Test, Sacral Thrust Test, Posterior Pelvic Pain Provocation (PPPP), and FABER Test administered by a physiotherapist to confirm diagnosis. The sample included both the primiparous and multiparous women. Battery tests:

1. ASLR–evaluates pelvic and lower limb stability by lifting one leg while supine (Liebenson et al., 2009).
2. The Pelvic Compression Test, involving compression of the pelvis, was used to test pain that was localized to the sacroiliac joints (Szadek et al., 2009)
3. Pelvic Distraction Test–applies outward pressure on the anterior pelvis to evaluate SI joint involvement (Werner et al., 2013).
4. Sacral Thrust Test–downward pressure on the sacrum to provoke SI joint pain (Telli, Telli & Topal, 2018).
5. Posterior Pelvic Pain Provocation–flexion of the hip to 90° with pressure along the femur to detect posterior pelvic pain (Östgaard, Zetherström & Roos-Hansson, 1994).
6. FABER Test–flexion, abduction, and external rotation of the hip to assess hip and sacroiliac joint dysfunction (Wong & Johnson, 2012).

### Exclusion criteria

Included postpartum symptoms such as nausea, dizziness, or blurred vision; high-risk pregnancies; structural spinal abnormalities (scoliosis, kyphosis, lordosis); traumatic, inflammatory, or infectious conditions; diagnosed psychological disorders; history of spinal, pelvic, or femoral surgery or fractures; neoplastic conditions; or prior episodes of back pain.

### Intervention

**Figure 2:**
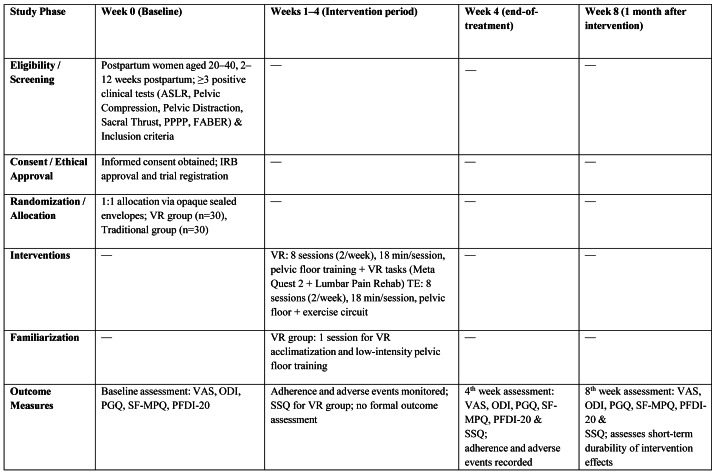
SPIRIT schedule of enrollment, interventions, and assessment.

Participants received eight physiotherapy sessions over four weeks, with two sessions per week. The duration and intensity of the sessions were gradually escalated and were described in the Procedure section. Adherence was monitored for each participant. Those subjects that missed two or more sessions in a row were regarded as dropouts. Reasons for missed sessions included personal scheduling conflicts or minor illness. The outcome measures, including primary pain indices (VAS), and secondary disability indices (ODI, PGQ, PFDI) were assessed at baseline, 4th week assessment (end-of-treatment), and 8th week assessment (1-month post-treatment). The study design is based on Standard Protocol Items: Recommendations for Interventional Trials (SPIRIT) 2013 explanation and elaboration, which guides protocols of clinical trials, with enrollment, allocation, intervention, and assessment structured according to SPIRIT principles. Shown in Fig. 2

### Procedure

#### TE as control group

An 18-minute training session was held that started with pelvic floor muscle training with 3 sets of 12 repetitions of 3 s of contraction and 3 s of relaxation in a lying position. The second part was be performed by with 2 sets of 5-minute training modules with 1-minute rest between modules. A five-exercise circuit was performed with one minute per exercise where the aim is to achieve the maximum number of repetitions possible, and then move on to the next exercise. For the exercises 2 dumbbells of 1 kilogram was required. The exercises included kegel pelvic floor exercise, dead weight lifts, wide back rows, lunges with rotation, good morning (hip hinge), and lateral side bends. These TE were designed to build strength and improve pelvic floor function, LPP mirroring (Ehsani et al., 2020).

#### VR intervention group protocol

Participants in the intervention group underwent a VR based exercise program using Meta Quest 2 head-mounted displays. The VR intervention consisted of eight structured sessions designed to address postpartum LPP through the immersive, interactive training. Each session incorporated pelvic floor muscle training using VR, consisting of three sets of 12 repetitions, with each contraction and relaxation phase lasting three seconds. In addition, patients engaged in two five-minute blocks of immersive VR activities with a two-minute rest interval, resulting in total session duration of 18 min.

A familiarization session was conducted before the intervention to acclimate participants to the VR environment. This included modified pelvic floor training (three sets of eight repetitions; 3 s of contraction and 3 s of relaxation), a low back pain VR module (History Mode, Level 1) featuring three minutes of gameplay in an asymmetrical foot-standing position with slow-speed bending movements at one-third of the range of motion (ROM), and one minute of reaction speed training involving two colours and one-second obstacle intervals.

Subsequent sessions progressively increased in intensity and complexity. Each session included standard pelvic floor training, followed by a low back pain module ranging from Level 2 to Level 9 in History Mode. These modules incorporated varying combinations of speed (slow, medium, fast), postural demands (asymmetrical stance), and movement patterns, including bending, extension, inclination, rotation, and tilt. The ROM also advanced from one-third to full capacity across the program. Each session concluded with two minutes of reaction speed VR training, maintaining a one-second response interval with two-colour stimuli and moving obstacles.

### Outcome measures

#### Primary outcome

Pain intensity was the primary outcome measured using the VAS (Begum & Hossain, 2019), ranging from 0 reporting no pain and 10 interpreted as worst pain.

#### Secondary outcomes

Multidimensional pain was assessed using the Short Form McGill Pain Questionnaire (SF-MPQ) (Dworkin et al., 2015) with a cut-off score of <34. The tool has 15 pain descriptors 11 sensory and four affective with a scale of 4 points to depict the intensity: 0 = none, 1 = mild, 2 = moderate and 3 = severe.

The Oswestry Disability Index (ODI) was used to measure functional disability (Fairbank & Pynsent, 2000) which is a 10-item, patient-reported questionnaire used to assess functional impairment from lower back pain in both acute and chronic conditions. Disability levels are categorized from minimal to crippling, with scores ranging from 0–100. Each item is scored from 0 to 5, yielding a total score with a cut of ≤50.

Pelvic floor dysfunction was evaluated using the Pelvic Floor Disability Index (PFDI-20) (Melkie et al., 2022) a 20-item questionnaire assessing the impact of bladder, bowel, and pelvic organ dysfunction on daily activities and quality of life. It includes three subscales: UDI-6, POPDI-6, and CRADI-8.

Pelvic girdle pain and functional limitations were assessed using the Pelvic Girdle Questionnaire (PGQ) (Stuge et al., 2011), a 25-item tool with two subscales: items 1–20 for daily activity restrictions and items 21–25 for symptom intensity (Melkie et al., 2022).

The Simulator Sickness Questionnaire (SSQ) assesses symptoms across three domains: nausea (N), oculomotor (O), which includes eye strain, blurred vision, and headache, and disorientation (D), which captures dizziness and vertigo. All the symptoms are rated using a 4-point Likert scale. To calculate SSQ scores, each subscale is weighted according to standard coefficients, with the sum of relevant items multiplied by 9.54 for nausea, 7.58 for oculomotor, and 13.92 for disorientation. The total SSQ score is then computed using the formula Total SSQ = 3.74 × (N + O + D), yielding a weighted score that typically ranges from 0 to approximately 200. Higher scores indicate greater severity of simulator-induced symptoms (Kennedy et al., 1993).

All instruments were used according to published licensing terms, with permission obtained from the respective copyright holders for academic use.

### Statistical analysis

SPSS version (IBM Corp., Armonk, NY, USA) 26 was used in all the analyses. The primary outcome was pain intensity measured using the VAS, for which the study was powered based on previous literature. Secondary outcomes, including functional disability (ODI, PGQ), pelvic-floor symptoms (PFDI-20 subscales), and multidimensional pain (SF-MPQ), as well as exploratory outcomes such as simulator sickness (SSQ), were considered hypothesis-generating and analyzed in an exploratory manner, as the study was not specifically powered to detect differences in these measures.

The Shapiro–Wilk test was used to determine the normality of continuous variables. VAS, SF-MPQ and SSQ scores were normally distributed and analyzed using parametric tests (independent t-tests for between-group comparisons and repeated-measures ANOVA for longitudinal analyses). Skewed variables (ODI, PGQ, PFDI-20) were analyzed using non-parametric tests (Mann–Whitney U) as appropriate. Between-group comparisons for repeated measures were performed using repeated-measures ANOVA, with baseline covariates including age, body mass index (BMI), mode of delivery, previous pelvic pain, and pre-delivery incontinence included in multivariate analyses to adjust for potential confounding. Adjusted group differences are reported with 95% confidence intervals.

Given the number of secondary and exploratory outcomes, *p*-values for these analyses were interpreted cautiously. All secondary and exploratory outcome analyses are considered hypothesis-generating and intended to inform future confirmatory studies. The primary outcome (VAS) remains the confirmatory endpoint. The compliance with the intervention of the participants was observed during the research. The main analyses were performed based on the concept of the intention-to-treat (ITT) principle, including randomized participants (*n* = 60). In cases where the study subjects dropped out before the 8th week assesment evaluation, they were assigned missing data points as the last observation carried forward (LOCF). Furthermore, a per-protocol sensitivity analysis excluding participants who missed ≥2 consecutive sessions was conducted to evaluate the strength of the results.

## Results

Four participants (two in each group) discontinued before the 8th week assessment due to personal scheduling conflicts or minor illness. The main analysis comprised all of the randomized subjects (*n* = 60) on the basis of the ITT principle, with the missing data on the 8th week substituted with the LOCF method. Dropout was minimal and balanced across groups, making major bias unlikely. A per-protocol sensitivity analysis excluding these participants yielded comparable results. The results of ITT and PP analyses were highly consistent, showing similar patterns of improvement in VAS, SF-MPQ, ODI, PGQ, PFDI-20 and SSQ scores, confirming the robustness of the intervention effects.

Table 1 shows the baseline demographic and clinical data were similar in both the VR (Group A) and TE (Group B) group. Age distribution did not differ between groups, with 36.7% of participants aged ≤28 years and 63.3% aged >28 years in both groups (*p* > 0.999). The body mass index was also evenly distributed across the study groups (*p* = 0.935). Pelvic pain at baseline (56.7–50.0 0.605) and pre-delivery urinary incontinence (6.7–6.7 0.999) also did not demonstrate any significant difference. However, a significant imbalance was observed in mode of delivery, with a higher proportion of cesarean sections in the VR group compared to the TE group (90.0% *vs* 66.7%, *p* = 0.028). To account for this imbalance, mode of delivery was included as a covariate in subsequent analyses, and its interaction with treatment group was examined.

**Table 1:** Comparison of baseline according to experimental and control group.

		**Group**			
**Baseline information**	**Categories**	**Group A**	**Group B**		***p*-value**
Age group	≤28 years	11 (36.7%)	11 (36.7%)	22 (36.7%)	>0.999
	>28 years	19 (63.3%)	19 (63.3%)	38 (63.3%)	
Body mass index	Normal	11 (36.7%)	12 (40.0%)	23 (38.3%)	0.935
	Overweight	13 (43.3%)	13 (43.3%)	26 (43.3%)	
	Obese	6 (20.0%)	5 (16.7%)	11 (18.3%)	
Pelvic pain			17 (56.7%)	15 (50.0%)	32 (53.3%)	0.605
Mode of delivery	SVD	3 (10.0%)	10 (33.3%)	13 (21.7%)	0.028
	C-Section	27 (90.0%)	20 (66.7%)	47 (78.3%)	
Incontinence prior to delivery			2 (6.7%)	2 (6.7%)	4 (6.7%)	>0.999

**Notes.**

Group A, VR group (Experimental), Group B TE (Control) group.

Table 2 compares outcome variables of the VR (Group A) and TE (Group B) groups at the baseline and 4th and 8th week assessments. There were no significant differences between groups in baseline measures of VAS, SF-MPQ, ODI (*p* = 0.631), PFDI (*p* = 0.401), and scores on pelvic girdle pain (*p* = 0.130).

**Table 2:** Comparison of primary and secondary outcomes variables according to study groups.

	Groups	Group A	Group B	*p*-value	Effect size
Scale					
VAS scale	Baseline	7.2367 ± 0.149	7.096 ± 0.129	0.847^†^	0.18
	4th week	5.1067 ± 0.1582	5.8033 ± 0.1459	0.002^†^	0.84
	8th week	3.2900 ± 0.1750	4.8000 ± 0.1591	<0.001^†^	1.48
Mc Gill SMPQ	Baseline	29.03 ± 1.61	29.07 ± 2.65	0.953^†^	0.02
	4th week	21.33 ± 3.01	19.20 ± 3.14	0.009^†^	0.32
	8th week	14.20 ± 2.89	19.73 ± 2.49	<0.001^†^	0.61
Oswestry disability index	Baseline	19.5 (10.75–28.25)	18 (8.75–27.25)	0.631^‡^	0.06
	4th week	11.50 (6.75–21.25)	10 (6.75–14)	0.299^‡^	0.13
	8th week	7 (4–8.25)	10 (4.00–10.25)	0.003^‡^	0.35
Pelvic floor disability	Baseline	175 (150–200)	162.50 (125–225)	0.401^‡^	0.11
	4th week	137.50 (100–150)	125.00 (100–150)	0.193^‡^	0.17
	8th week	100 (7–100)	75.00 (75–100)	0.72^‡^	0.02
Pelvic girdle pain	Baseline	47 (44–50)	50 (46–50)	0.13^‡^	0.20
	4th week	37.5 (34.5–41)	41 (39–44.25)	<0.001^‡^	0.46
	8th week	31.5 (30–35)	38.5 (35–40)	<0.001^‡^	0.59
Simulator sickness	Baseline	7.79 ± 1.80	Not applicable	(within-group)	
	4th week	4.34 ± 2.11		0.001^§^	0.658
	8th week	3.19 ± 2.28			

**Notes.**

VAS in both groups was statistically significantly lower than the previous Time (*p*-value < 0.001).

† Independent *t*-test (parametric).

‡ Mann–Whitney U test (non-parametric).

§ Repeated-measures ANOVA.

Across 4th and 8th week assessments, Group A demonstrated significantly greater reductions in VAS pain scores compared with Group B. Differences between groups were significant at the 4th week (*p* = 0.002) and further increased by the 8th week (*p* < 0.001). The magnitude of pain reduction observed in the VR group exceeded the established minimal clinically important difference (MCID) of 1.5–2.0, indicating clinically meaningful pain improvement over the course of the intervention.

There was no significant difference between groups in ODI at the 4th week (*p* = 0.299), but this was significant at the 8th week with higher improvement in Group A (*p* = 0.003). The overall improvement in the VR group exceeded the ODI MCID of 10 points, reflecting a clinically important reduction in disability by the end of the intervention period.

At the 4th week (*p* < 0.001) and the 8th week assessment (*p* < 0.001), Group A exhibited considerably more reductions in the scores of PGQ as compared to Group B. When considered across 4th and 8th week assessments, the magnitude of change in the VR group fell within the published MCID range of 10–15% of the maximum PGQ score, indicating clinically meaningful improvement in pelvic girdle–related symptoms.

For other outcomes, SF-MPQ scores differed between groups at 4th and 8th week assessments, favoring Group B at the 4th week and Group A at the 8th week assessment, while no significant between-group differences were observed for PFDI at either follow-up.

Simulator Sickness Questionnaire (SSQ) scores in the VR group decreased progressively over time, from 7.79 ± 1.80 at baseline to 4.34 ± 2.11 at the 4th week and 3.19 ± 2.28 at the 8th week assessment, indicating reduced simulator-related discomfort across sessions. Repeated-measures ANOVA revealed a significant within-group effect of time (*p* < 0.001) with a large effect size (partial *η*^2^ = 0.658), indicating substantial reduction in simulator-related symptoms over time.

A two-way ANOVA was conducted to examine the effects of intervention group (VR *vs* TE) and mode of delivery (cesarean *vs* vaginal) on VAS scores at the 8th week assessment. There was a significant main effect of group (F (1,56) = 26.34, *p* < 0.001, *η*^2^*p* = 0.32), but no significant main effect of mode of delivery (F (1,56) = 0.006, *p* = 0.938, *η*^2^*p* = 0.000). The interaction between group and mode of delivery was not significant (F (1,56) = 2.94, *p* = 0.092, *η*^2^*p* = 0.05), indicating that the intervention effect was consistent across delivery modes.

Table 3 shows subscale scores of the PFDI-20 (POPDI-6, CRADI-8, and UDI-6) at baseline, 4th and 8th week assessments. No significant between-group differences were observed at any time point, indicating that both VR and TE improved pelvic floor symptoms similarly. The VR intervention, as a whole, demonstrated better outcomes in the intensity of pain, sensory-motor pain, disability, and pain in the pelvic girdle compared to TE, with no significant difference observed in changes in the pelvic floor disability.

**Table 3:** Comparison of PFDI-20 subscale scores between Group A and Group B across follow-up periods.

Subscale of PFDI-20		Group A	Group B	*p*-value
POPDI-6	Baseline	57.50 ± 11.65	59.17 ± 12.25	0.587
	4th week	40.00 ± 12.46	38.33 ± 12.68	0.605
	8th week	25.83 ± 4.56	25.83 ± 4.56	1.000
CRADI-8	Baseline	60.83 ± 12.60	53.33 ± 23.43	0.308
	4th week	34.17 ± 12.25	31.67 ± 11.24	0.409
	8th week	30.83 ± 10.75	30.83 ± 10.75	1.000
UDI-6	Baseline	60.00 ± 12.46	60.00 ± 12.46	1.000
	4th week	52.50 ± 16.54	52.50 ± 16.54	1.000
	8th week	35.00 ± 12.46	36.67 ± 14.28	0.718

Table 4 presents the multivariate analyses examining potential predictors of outcome measures. Age, mode of delivery, and pre-delivery incontinence did not significantly predict any of the outcomes, including ODI, VAS, SF-MPQ, SSQ, and PFDI-20, as all *p*-values were > 0.05. Mode of delivery was not a significant predictor of any outcome measure and did not alter the estimated treatment effects. BMI was a significant predictor only for ODI (*F* = 6.86, *p* = 0.011, *η*^2^*p* = 0.113), indicating that higher BMI was associated with greater disability. Before the intervention, pelvic pain was a significant predictor of SSQ scores (*F* = 8.01, *p* = 0.007, 52p = 0.129); thus, participants who complained of pelvic pain at baseline had higher simulator sickness. On the whole, of the variables studied, only BMI and baseline pelvic pain were found to be significant predictors, with age, mode of delivery, and previous incontinence not having significant impacts on the measured results.

**Table 4:** Multivariate analyses of predictors for outcome variables.

**Predictors**	**Dependent variable**	***F***	**Sig.**	***η* ^2^ *p***
Age	ODI	1.19	0.28	0.022
	VAS	2.306	0.135	0.041
	McGill	0.05	0.825	0.001
	SSQ	0.01	0.906	0
	PFDI-20	2.69	0.107	0.048
BMI	ODI	6.86	0.011	0.113
	VAS	0.076	0.784	0.001
	McGill	2.97	0.09	0.052
	SSQ	0.45	0.506	0.008
	PFDI-20	1.34	0.253	0.024
Mode of delivery	ODI	0.63	0.429	0.012
	VAS	0.251	0.619	0.005
	McGill	0.2	0.656	0.004
	SSQ	2.81	0.099	0.049
	PFDI-20	3.79	0.057	0.066
Pelvic pain	ODI	1.84	0.18	0.033
	VAS	1.924	0.171	0.034
	McGill	1.37	0.248	0.025
	SSQ	8.01	0.007	0.129
	PFDI-20	1.55	0.218	0.028
Incontinence prior delivery	ODI	0.91	0.346	0.016
	VAS	0.014	0.907	0.000
	McGill	0.89	0.351	0.016
	SSQ	0.14	0.708	0.003
	PFDI-20	1.78	0.188	0.032

**Notes.**

F, F-statistic; Sig., *p*-value; *η*^2^*p*, partial eta squared (effect size). *p* < 0.05 indicates statistical significance.

## Discussion

The present study examined the impact of VR based rehabilitation program on postpartum LPP, disability, and pelvic floor dysfunction, compared to a TE program. Findings revealed that the VR group achieved significantly greater improvements in pain intensity *i.e.,* VAS, disability in ODI scale, and pelvic pain by the PGQ across both assesments. While both groups achieved similar recovery in terms of prolapse and urinary symptoms, the control group showed slightly better relief of bowel symptoms as measured by PFDI-20. These results reflect short-term benefits of VR-based rehabilitation, as follow-up was limited to one month, and the sustainability of these improvements over the long term cannot be determined.

The current research shows a significant reduction in pain within the VR group (VAS scores dropping from 7.23 ± 0.149 to 3.29  ± 0.175, *p* = 0.847 and *p* < 0.001 at subsequent 4th and 8th week assessment), accompanied by a large effect size (*d* = 1.48). The magnitude of this change exceeds the established MCID of 1.5–2.0 on the VAS Farrar et al., 2001), indicating a clinically meaningful reduction in pain and supporting the effectiveness of VR-based rehabilitation for pain management. The immersive VR tasks likely served as powerful attentional distractions, engaging brain regions involved in pain modulation, such as the prefrontal cortex and anterior cingulate cortex, and activating descending inhibitory pathways that reduce nociceptive signaling (Hoffman et al., 2007). These neurophysiological mechanisms are less effectively stimulated by the control group’s TE, which primarily focuses on physical strengthening without immersive distraction or cognitive engagement (Srinivas et al., 2021). The marked increase in participants reporting no pain with *p* < 0.001 at the 8th week assessment further supports VR’s analgesic benefits, consistent with findings from Rezai et al. (2023), who reported similar pain reductions and improvements in patient satisfaction among VR users during laceration repair in the emergency department. A systematic review and meta-analysis by Mallari et al., 2019), synthesizing data from 20 randomized controlled trials, related to acute and chronic pain in conditions like burns, musculoskeletal, neuropathic, and other medical procedures reveled the effect of VR most prominently in acute pain rather than chronic pain, where people did not use VR for a long time to narrate the long lasting effects of VR. Afzal et al. (2022) indicated a more pronounced decrement in VAS within the VR cohort (from 6.62 ± 1.04 and 1.00 ± 0.60) when compared to the TE group (from 6.62 ± 1.04 to 3.32 ± 0.81; 39%) in mixed gender with chronic low back pain. In a study comparing the effect of VR and core stability exercise in chronic low back pain in collegiate athletes by Abdelraouf et al. (2020) revealed that VR combined with core stability exercises revealed promising results in alleviating chronic LBP, which alone with one of the treatment won’t give better promising. These results indicate that VR (only) might not lead to any long-term neuroplastic effect needed in managing chronic pain, especially in complicated pain such as postpartum LPP. In our research, VR resulted in premature alleviation of pain perception, which was probably caused by immersive distraction, decreased threat perception, and increased early self-efficacy in postpartum women. However, as follow-up was limited to one month, the sustainability of these benefits beyond the short term remains unknown, and further research with longer follow-up periods is needed to evaluate lasting effects.

The current research noted that the SF-MPQ scores reduced significantly in both the intervention groups, and this implies that the characteristics of pain decreased. The VR group, in its turn, improved more. This corresponds to the findings of a study conducted by Trujillo et al. (2020), who assumed that embodiment in VR could be used to treat the symptoms of chronic LBP through reinterpreted sensory feedback of the body. The current research study has indicated that the decrease in the intensity of the pain was higher in the VR group than in the TE group. This finding is also supported in a study by García-López et al. (2023) that the program of VR and physiotherapy proved to be more productive than the usual physiotherapy in the perception and prevention of pain in pregnant women with LBP and pelvic pain. These results show that VR is capable of demonstrating a new approach to rehabilitation in contrast to the TE. However, a study by Saleh, Botla & Elbehary (2019) stated that the level of pain among postpartum women had improved dramatically after the application of core stability exercises. Moreover, a systematic review conducted by Moheboleslam, Mohammad Rahimi & Aminzadeh (2022) discovered that stabilizing exercises could be used to decrease the severity of pain in postpartum women with LPP. The current research noted that SF-MPQ scores decreased significantly in both intervention groups, indicating reduced pain characteristics, with the VR group showing greater improvement with a small effect size (*d* = 0.32) in the 4th week, accompanied by a moderate effect size (*d* = 0.61) in the 8th week assessment. These findings suggest that VR can provide an innovative approach to postpartum rehabilitation, complementing TE programs. However, as follow-up assessments were limited to one month, the durability of these pain reductions beyond the short term cannot be determined. Future studies with longer follow-up periods are needed to evaluate whether VR interventions produce sustained improvements in musculoskeletal pain associated with postpartum LPP.

This study supports the growing evidence that VR exhibited significantly greater improvements in disability scores compared to the TE group, with benefits persisting across the assessments accompanied by a moderate effect size (*r* = 0.35) at 8th week. The magnitude of improvement in the VR group exceeded the established ODI MCID of 10 points (Ostelo et al., 2008), indicating that the reductions in disability were not only significant but also clinically meaningful. The immersive VR training, combined with pelvic floor contractions, likely contributed to this improvement by providing multisensory feedback and dynamic motor challenges that facilitated enhanced neuromuscular control of the lumbopelvic region (Li et al., 2024). In contrast, the TR group’s conventional exercise protocol, while beneficial, provided less varied stimuli and may have led to slower neuromuscular adaptation. This is reflected in the relatively modest change in ODI scores, with median values remaining largely stable from the 4th week to the 8th week assessment, indicating limited additional reduction in disability over time. Huang & Chen (2025) demonstrated in a systematic review of 15 randomized control trial of 711 patients that VR therapy led to a markedly greater improvement in ODI scores compared to conventional care in chronic LBP. Another study by Kim et al. (2014) on middle-aged women for chronic LBP showed significantly improved disability on the ODI scale through a VR-based yoga program. A study conducted by Afzal et al. (2023) on mixed gender populations regarding chronic LBP shows promising results in the low back disability index using VR as compared to back-strengthening exercises. Notwithstanding these encouraging findings, there exists contradictory evidence. Opara, Kozinc & Ivezić (2024) did not establish VR-based rehabilitation as superior to TE for enhancing ODI in postpartum women, as the investigation concentrated on general chronic musculoskeletal pain (12.4 ± 2.5 *vs.* 5.3 ± 3.7). Likewise, Chen et al. (2024) scrutinized no significant enhancement in ODI of VR therapy (−3.1 points *versus* control: −2.7 ± 1.55). Stamm (2024) similarly documented no notable benefit of VR exergames *vs.* standard care therapy (−6.4 ± 3.77 *versus* −5.9 ± 2.14). A study by Gokcen et al. (2022) found that TE were found to have a better positive effect on ODI scores than home-based programs, with the mean difference of 14.2 (95% CI [−18.1 to −10.3]). In Stuge et al. (2004) studies, there was a higher disability reduction through traditional and core stability exercises. VR, which provides immersive gamified therapy, improves neuromuscular control and core coordination and has shown the promise of improving the pain and disability outcomes of various musculoskeletal conditions (Ciubean et al., 2024). Notwithstanding these encouraging findings, it is important to note that follow-up was limited to one month, and the persistence of disability improvements beyond this short-term period cannot be determined. Although VR seems to have significant short-term benefits in terms of postpartum disability, additional research, including follow-up observations, is required to estimate the long-term benefits.

This study found significant improvements in women’s quality of life related to PGQ in the VR group, with PGQ scores improving at both 4th and 8th week assessments. Although both groups showed improvement over time, the VR group demonstrated greater reductions, indicating a moderate effect size *r* = 0.46 at the 4th week and representing a large effect size *r* = 0.59 at the 8th week, compared with the TE group, respectively. The magnitude of improvement in the VR group exceeded the published MCID for PGQ (10–15% of the maximum score) (Stuge, Jenssen & Grotle, 2017), indicating that these changes were not only significant but also clinically meaningful. The VR exercises offer multisensory inputs and real-time feedback, which improve the neuromuscular coordination and the pelvic-lumbar muscle activity. This enhances motor control, decreasing compensatory movements and biomechanical load, and can renew impaired neuromotor pathways through training and respond to significant dysfunctions related to the quality of life of postpartum women. These results are consistent with the recent studies conducted by Tayyebi et al. (2025), who determined that balance, function, and psychological well-being of Parkinson patients improved. Sekar et al. (2024), who identified that balance and mobility improvements were greater in elderly patients using VR; and Lin, who found VR to be a useful tool to improve the quality of life of women in various clinical conditions (Hou et al., 2020). However, as follow-up assessments were limited to one month, the persistence of these improvements in quality of life beyond the short term cannot be determined. These results indicate that VR exercises can be useful with a short-term positive effect to postpartum women experiencing PGQ, but the long-term effect of this type of exercise needs to be considered in future studies.

The symptoms of pelvic organ prolapse (POPDI-6), bowel dysfunction (CRADI-8), and urinary symptoms (UDI-6) were assessed using PFDI-20 subscales. Both VR and TE groups achieved moderate improvement by the 8th week assessment. For prolapse symptoms (POPDI-6), VR and TE groups improved from baseline to 8th week assessment, reflecting substantial symptom reduction. Bowel dysfunction (CRADI-8) improved in both groups by the 8th-week assessment, with slightly greater improvement observed in the TE group compared to the VR group. Urinary symptoms (UDI-6) decreased in both groups by the 8th-week assessment, showing comparable improvements between the VR and (TE) groups. The overall between-group effect size for PFDI-20 at the 8th week was negligible (*r* = 0.02), indicating that while both interventions effectively reduced symptoms, the magnitude of difference between FIVR and TE was minimal. All improvements in prolapse, bowel, and urinary symptoms reflect participant self-reported outcomes measured using the PFDI-20 subscales; no clinician-assessed anatomical evaluation was performed .Both interventions included pelvic floor contractions, but the TE group’s additional focus on targeted resistance exercises, such as kegel and lunges likely contributed to effective muscle strengthening and functional recovery, which was reflected in comparable symptom relief in prolapse and urinary functions. The VR group’s pelvic floor engagement, integrated within the dynamic VR tasks, may have provided an indirect but effective means of muscle activation. Nonetheless, bowel symptoms improved slightly more in the TE group, suggesting that specific pelvic floor symptoms linked to autonomic or sphincter control might respond better to traditional, focused muscle training than the more generalized neuromuscular stimulation of VR. Rutkowska et al. (2022), who reported that VR based pelvic floor muscle training improved urinary symptoms and muscle function, but outcomes were similar to traditional therapy. García-López et al. (2023) also showed that VR combined with physiotherapy improved pain and function during pregnancy. Despite these positive findings in the above researches the field remains in its early stages, and more robust research is needed to determine if VR offers benefits in pelvic floor dysfunction symptoms. However, some pelvic floor functions directly related to autonomic or sphincteric control can be better treated using standard targeted exercises, and it is clear that future research is necessary to refine VR programs in these areas. As with other outcomes, follow-up in the current study was limited to one month, so the long-term sustainability of pelvic floor symptom improvements remains unknown. From a pathophysiological perspective, this may reflect differential neuromuscular recruitment and reflex pathways that require specific training stimuli not fully replicated in current VR programs (Hodgson, 2025).

The Simulator Sickness Questionnaire (SSQ) was used to assess VR-related discomfort in the experimental group. Mean SSQ scores decreased at the 8th week assessment, representing an approximate 59% reduction over time. In VR research, total SSQ scores below 10 are generally interpreted as minimal to mild symptoms, whereas scores exceeding 20 are considered indicative of clinically relevant simulator sickness. Therefore, participants demonstrated a shift from mild baseline symptoms to minimal levels with repeated exposure. Although no universally established MCID exists for SSQ, large effect size (partial *η*^2^ = 0.658) indicate substantial symptom reduction. Previous VR research has shown that SSQ is a valid and sensitive measure of simulator sickness in immersive VR applications, with higher total SSQ scores reflecting greater discomfort across studies (Sevinc & Berkman, 2020). Additionally, systematic evidence on HMD-based VR contexts demonstrates that VR sickness profiles and corresponding SSQ scores vary with exposure and user adaptation, and tend to decrease with repeated use (Saredakis et al., 2020). In line with this, other VR training studies have found that SSQ scores improve with continued exposure, reflecting adaptation to the virtual environment (Takata et al., 2021). These thresholds are based on general VR literature and may not be specific to postpartum populations. The control group was not assessed with SSQ, as they were not exposed to VR. Overall, the improvement in the VR group supports the safe implementation of VR-based rehabilitation.

The research was carried out in one center within Pakistan, which restricted the generalization of the findings. Participants were postpartum women in a specific socioeconomic and healthcare context, which may differ from populations in high-income countries where VR adoption, cost, and accessibility are higher. The vast majority of research conducted in VR rehabilitation is focused on high-income contexts, and comparatively few studies consider low-income and middle-income countries (Pawassar & Tiberius, 2021). Therefore, results should be interpreted cautiously when applying to other populations. More research should be conducted to apply VR-based interventions in different environments to determine the feasibility, cost, and the applicability of this intervention in various parts of the world.

## Limitation of the Study

Evidence supporting VR for musculoskeletal pain is largely derived from acute or chronic conditions and pregnancy or labor, with limited postpartum-specific data, making these findings exploratory. Participant blinding was not feasible, potentially introducing performance bias and a novelty effect. The intervention consisted of eight sessions over one month with a single one-month follow-up, limiting assessment of long-term effectiveness. The sample size (*N* = 60) is very limited and does not allow generalization. Moreover, the hospital-based VR equipment and costs might restrict feasibility, availability, and sustainability in the standard clinical applications.

## Future Lines of Research

Long-term randomized controlled trials involving larger and more diverse samples to determine VR benefits sustainability over 6–12 months and enhance generalizability should be incorporated in further research. Studies should also explore the feasibility and effectiveness of home-based VR programs using user-friendly systems and remote monitoring to enhance accessibility. Formal cost-effectiveness analyses comparing VR based rehabilitation with standard physiotherapy are needed to determine economic viability. Moreover, the research should be conducted to explore the hidden mechanics of VR by integrating objective, biomechanical and neuromuscular outcomes. Finally, adaptation of VR protocols for other perinatal populations and related conditions may broaden its clinical applications.

## Clinical Applications

The VR eight-session program demonstrates potential as an adjuvant to postpartum rehabilitation, especially in the areas of LPP and pelvic floor dysfunction. It has an immersive functional design, which increases adherence, core stability, and neuromuscular control, particularly with patients who are kinesiophobic or bored with exercise. While initially supervised in clinical settings, VR could be adapted for home use to increase accessibility. In addition to the postpartum care, VR can be used to complement rehabilitation in chronic LBP, post-operative recovery, and balance training of older adults. VR can enhance patient engagement, treatment effectiveness, and utilization of clinical resources by allowing them to engage in the management to an extent, offer objective feedback, and decrease supervision requirements.

## Conclusion

The findings of this study indicate that VR-based rehabilitation resulted in greater short-term improvements in pain intensity and disability compared with TE alone across follow-up assessments. By the 8th week assesment, participants in the VR group demonstrated clinically meaningful reductions in pain and disability, while between-group differences in functional capacity and quality of life were modest. However, as follow-up was limited to one-month post-intervention, the sustainability of these benefits beyond the short term cannot be determined. Overall, these results suggest that VR may serve as a promising adjunct to conventional management of postpartum LPP in the short term. The research needs to be extended with longer periods of follow-up to assess long-term efficacy, feasibility, and implementation issues, such as cost and access.

## Supplemental Information

10.7717/peerj.21202/supp-1 Supplemental Information 1 Raw Data

10.7717/peerj.21202/supp-2 Supplemental Information 2 CONSORT checklist

10.7717/peerj.21202/supp-3 Supplemental Information 3 Two-way ANOVA examining effects of intervention group and mode of delivery on VAS at 8th week Assessment

## Additional Information and Declarations

### Data Availability

The following information was supplied regarding data availability: The raw data are available in the Supplemental File.

### Clinical Trial Registration

The following information was supplied regarding Clinical Trial registration: NCT05921747.
