# Supplementary material for: The impact of virtual reality exercise programs on postpartum pelvic pain and disability among women with lumbopelvic pain
Source: PeerJ. 2026 Jul 1;14:e21202. doi: 10.7717/peerj.21202 (PMC13332716; doi:10.7717/peerj.21202)
Supplement: Supplementary file 1 [file peerj-14-21202-s002.odt]

# CONSORT Checklist

Title of Study: The impact of virtual reality exercise programs on postpartum pelvic pain and disability among women with lumbopelvic pain.

Trial registration: ClinicalTrials.gov Identifier: NCT05921747

Ethical Approval: Shalamar Medical and Dental College IRB (No.0548 / Ref: SMDC-IRB/AI/18-1/2023)

Study Design: Single-blind, parallel-group randomized controlled trial (1:1 allocation)

Study Setting: Gynecology Department, Shalamar Hospital, Lahore, Pakistan

Participants: 60 postpartum women aged 20–40 years with clinically confirmed lumbopelvic pain

 

| Section/Topic | Item No. | Checklist item | Reported on page/section |
| --- | --- | --- | --- |
| Title | 1a | Identification as a randomized trial in the title | Pg.#1 |
| Abstract | 1b | Structured summary of trial design, methods, results, and conclusions | Pg.# 2-3 |
| Introduction | 2 | Scientific background and explanation of rationale | Pg. # 4-5 |
| Methods | 3 | Study Design and ethical consideration, clinical trial,      sample size calculation, participants | Pg. # 6-8 |
| Procedure | 4 | Traditional exercise was used as the control group and the VR interventional group protocol. Detailed description of interventions for each group with sufficient detail to allow replication | Pg. # 9-10 |
| Outcomes | 5 | The use of 05 scales for the research.i.e. ODI,PFDI-20, PGQ, SF-MPQ, VAS | Pg. # 11-12 |
| Statistical Analysis | 6 | Statistical methods used to compare groups for primary and secondary outcomes | Pg. # 13 |
| Results – | 7 | Numbers assigned, treated, and analyzed | Pg. # 14-16 |
| Discussion | 8 | Trial limitations, sources of bias, imprecision and conclusion | Pg. # 17-24 |
| Other Information: | 9a | Registration number and name of trial registry | Abstract & Methods – NCT05921747 |
| Authors contribution | 9b |  | Submitted as Supplementary File |
|  | 9c | Where the full trial protocol can be accessed | Submitted as Supplementary File |
|  | 10 | Sources of funding and role of funders | Self-funded; stated in the Methods section |
| Consort flow diagram | 11 | Describe all steps of research | Submitted as Supplementary File |
| References | 12 |  | Pg. #25-28 |
| Annexure: | 12 |  | Submitted as Supplementary File |
