# Supplementary material for: The impact of virtual reality exercise programs on postpartum pelvic pain and disability among women with lumbopelvic pain
Source: PeerJ. 2026 Jul 1;14:e21202. doi: 10.7717/peerj.21202 (PMC13332716; doi:10.7717/peerj.21202)
Supplement: Supplementary file 2 [file peerj-14-21202-s003.odt]

Two-way ANOVA examining effects of intervention group and mode of delivery on VAS at 8th week Assesment.”

| Source | F | df | p | Partial η² |
| --- | --- | --- | --- | --- |
| Group | 26.342 | 1,56 | <.001 | 0.320 |
| Mode of Delivery | 0.006 | 1,56 | .938 | 0.000 |
| Group × Mode of Delivery | 2.939 | 1,56 | .092 | 0.050 |

Note: F = F-statistic; Sig. = p-value; η²p = partial eta squared (effect size). p < 0.05 indicates statistical significance.
